# New Vision for Visual Prostheses

**DOI:** 10.3389/fnins.2020.00036

**Published:** 2020-02-18

**Authors:** Alexander Farnum, Galit Pelled

**Affiliations:** ^1^Department of Biomedical Engineering, College of Engineering, Michigan State University, East Lansing, MI, United States; ^2^Institute for Quantitative Health Science and Engineering, Michigan State University, East Lansing, MI, United States; ^3^Department of Radiology, College of Human Medicine, Michigan State University, East Lansing, MI, United States

**Keywords:** vision, bioengineering, visual prostheses, neuromodulation, magnetic stimulation, cortical implant

## Abstract

Developments of new strategies to restore vision and improving on current strategies by harnessing new advancements in material and electrical sciences, and biological and genetic-based technologies are of upmost health priorities around the world. Federal and private entities are spending billions of dollars on visual prosthetics technologies. This review describes the most current and state-of-the-art bioengineering technologies to restore vision. This includes a thorough description of traditional electrode-based visual prosthetics that have improved substantially since early prototypes. Recent advances in molecular and synthetic biology have transformed vision-assisted technologies; For example, optogenetic technologies that introduce light-responsive proteins offer excellent resolution but cortical applications are restricted by fiber implantation and tissue damage. Other stimulation modalities, such as magnetic fields, have been explored to achieve non-invasive neuromodulation. Miniature magnetic coils are currently being developed to activate select groups of neurons. Magnetically-responsive nanoparticles or exogenous proteins can significantly enhance the coupling between external electromagnetic devices and any neurons affiliated with these modifications. The need to minimize cytotoxic effects for nanoparticle-based therapies will likely restrict the number of usable materials. Nevertheless, advances in identifying and utilizing proteins that respond to magnetic fields may lead to non-invasive, cell-specific stimulation and may overcome many of the limitations that currently exist with other methods. Finally, sensory substitution systems also serve as viable visual prostheses by converting visual input to auditory and somatosensory stimuli. This review also discusses major challenges in the field and offers bioengineering strategies to overcome those.

## Introduction

The development of bioelectrical interfaces in the 18th century enthralled scientists looking for strategies to treat brain pathologies and restore vision. Revolutionary experiments by LeRoy (1755) and Volta in 1800 (Volta and Banks, 1800) succeeded in demonstrating that electrical stimulation of the eye could produce spots of light, or phosphenes, in one’s visual field.

Since then, advancements in neuroimaging, electrophysiology hardware, and surgical equipment have spurred ground-breaking research uncovering intricacies of visual pathways and possible therapeutic targets. Visual prostheses allowing for the restoration of basic abilities promoting object discrimination (Stingl et al., 2013) and simple mobility (Humayun et al., 2012), are now viable therapeutic considerations for visually impaired and blind individuals. A few retinal-based prosthetic devices have already been approved for commercial use in Europe (Humayun et al., 2012; Hornig et al., 2017), one of which is also approved in the United States (Luo and Da Cruz, 2016).

This review describes state-of-the-art electrode-based visual prostheses technologies, and the ongoing development of cutting-edge biological- and genetic-based technologies to restore visual function including nanoparticles, optogenetics, magnetic manipulation and sensory substitution systems. These methods have the capability of artificially encoding sensation i.e. “writing,” into the brain and are gaining considerable interest as next-generation visual therapeutics.

In 2015, there were 253 million individuals (3.43% of global population) blind and moderately-to-severely visually impaired people in the world (Bourne et al., 2017). By 2050, owing to a dramatic increase in life expectancy, it is predicted that this number will rise to 703 million (7.19% of global population) (Bourne et al., 2017). Blindness is the most feared condition by the American public, more so than Alzheimer’s disease, cancer and HIV/AIDS (Scott et al., 2016). Visual deficits are strongly associated with economic (Wittenborn et al., 2013), physical (McLean et al., 2014; Crews, 2016), and emotional (Stelmack, 2001; Hassell et al., 2006) detriments. While population aging is still in its early stages, large-scale communities and nations are already being challenged by the increased medical and fiscal responsibilities associated with visual impairments (Gordois et al., 2012; Wittenborn et al., 2013). In fact, the majority of public health specialists have underestimated the rapidity of this epidemiological transition and the associated need for resource reallocation (World Health Organization, 2006). Thus, it is necessary to identify new interventions to address the steep increase of visual deficits among the world population.

## Electrode-Based Visual Prostheses

The traditional electrode-based visual prostheses consists of a basic set of components. A video camera is often used to convert light into electrical signals. These analog signals are digitized, and the image is processed by a portable micro-computer. The signals are then wirelessly transmitted to internal componentry with accompanying multi-electrode arrays (MEAs), which directly interface with the neural tissue.

The type of the electrode-based prostheses is dictated by the underlying pathophysiology and its location should target a region along the visual pathway that would be the most effective in restoring visual perception. Interfacing too early along the visual pathway could lead to either non-transmitted or significantly corrupted and unintelligible signals. Interfacing at a region later than necessary bypasses functional neuronal circuitry, requiring additional hardware and/or complex image-processing algorithms. There are four major prosthetic design-types, each of which are categorized based on the location of their associated MEAs. Table 1 summarizes the advantages and limitations of the different visual prostheses and their location.

**TABLE 1:** Advantages and limitations of different visual prostheses modalities with feasible interface locations.

**Intervention Type**	**Interface Location**	**Advantages**	**Limitations**	**References**
Electrode	Retinal Optic Nerve LGN Cortex	Efficient surgical implantation procedures Large pool of past research Biocompatible-material coatings Accessibility to deeper brain regions	Limited hermetic encapsulation Tissue and cell damage Limited spatial resolution Invasive Wireless telemetry for external hardware communication limits data transfer	Brindley and Lewin, 1968; Humayun et al., 1996; Schmidt et al., 1996; Veraart et al., 1998; Brelén et al., 2005; Pezaris and Reid, 2007; Zrenner et al., 2010; Panetsos et al., 2011; Da Cruz et al., 2013; Stingl et al., 2013; Lowery et al., 2017; Troyk, 2017; Pouratian et al., 2019
Optogenetics	Retinal Cortex	Excellent spatial resolution Excellent temporal resolution Cellular excitation or inhibition Non-invasive stimulation (retinal only) Cell specificity	No accessibility to deeper brain regions Limited cortical accessibility Phototoxicity possibility Tissue damage (cortical only) Invasive (cortical only) Need for high-power light source(s) Potential immune response	Waldvogel et al., 2000; Boyden et al., 2005; Bi et al., 2006; Chow et al., 2010; Lin et al., 2013; Reutsky-Gefen et al., 2013
Magnetic stimulation	Cortex	Non-invasive stimulation No introduction of exogeneous agents	Limited spatial resolution Limited resolution for deeper brain regions Need for high-power electromagnetic device(s)	Barker et al., 1985; Bonmassar et al., 2012; Park et al., 2013; Lee and Fried, 2016; Lee et al., 2016
Magnetic nanoparticles	Cortex	Non-invasive stimulation Cell specificity	Limited spatial resolution Limited resolution for deeper brain regions Potential cytotoxic or immune response Delivery to brain can disrupt blood–brain barrier	Hughes et al., 2007; Huang et al., 2010; Baid et al., 2013; Chen et al., 2015; Guduru et al., 2015; Munshi et al., 2018
Genetically encoded magnetic stimulation	Cortex	Non-invasive stimulation Cell specificity	Limited spatial resolution Limited resolution for deeper brain regions Potential immune response	(Wheeler et al., 2016; Krishnan et al., 2018)
Sensory substitution	Periphery	Non-invasive Suitable for any visual ailment	Limited spatial resolution Occupies another key sensory modality Requires additional training	Bach-y-Rita et al., 1969, 1998; Meijer, 1992; Chebat et al., 2007; Striem-Amit et al., 2012; Abboud et al., 2014

### Retinal

Retinal prostheses offer promising rehabilitative potential for a number of retinal-based pathologies, including retinitis pigmentosa and age-related macular degeneration. In 1956, Tassicker developed and implanted the first retinal prostheses, capable of providing the recipient with crude light perception (Tassicker, 1956). Nearly 40 years later, Humayun et al. demonstrated that focal electrical excitation of the retinal surface could elicit cortical responses in animal models (Humayun et al., 1994) and localized visual percepts in human patients (Humayun et al., 1996). These preliminary experiments paved the way for retinal implants as the most common visual prostheses due to the orderly retinotopic organization, ease of surgical accessibility, and early positioning in the visual pathway. Two retinal prostheses sub-types, subretinal and epiretinal, constitute the majority of retinal prostheses-based research.

In the fovea, the most visually acute portion of the retina, there are an average of 150,000 (Shroff, 2011) and a peak density of 200,000 cone photoreceptors per mm^2^ (Curcio et al., 1990). The diameters of cone outer segments and retinal ganglion cells are 3–5 μm and 6–13 μm, respectively (Hebel and Holländer, 1983; Shepherd, 2003). Although improvements in micromachining and lithography now allow for the development of precise electrode arrays, targeting each neuron independently to reproduce natural vision is still challenging. Fortunately, extrapolating from cochlear implant patients, functional vision restoration most likely requires a total electrode count that is a mere fraction of the number of retinal neurons. Moreover, patients have been observed to demonstrate a significant learning effect and task-based improvements shortly after implantation.

#### Subretinal

Retinal-based diseases are characterized by photoreceptor cell death that glasses and contact lenses, which only refocus light rays through the cornea and lens, cannot address. Subretinal prostheses allow for the earliest possible intervention in the visual process. The prostheses most often consists of metallic electrode contacts embedded in a biocompatible polymeric film. It is positioned within the largely degenerated photoreceptor layer and directly interface with retinal bipolar cells. Since they interact with outer-retinal tissue, subretinal prostheses retain substantial intra-retinal signal processing. This allows for the generation of more naturalistic phosphenes, serving to expedite patient training periods relative to other types of visual prostheses.

Some subretinal implants even do away with the need for an external imager altogether via the use of microphotodiode arrays (MPDAs). Early research has shown that photodiodes reliant solely on ambient light can induce neurotrophic effects, but are insufficient for phosphene generation (Chow et al., 2004; Palanker et al., 2005). By incorporating circuitry for signal amplification, significant responses were seen in animal models (Lorach et al., 2015; Prévot et al., 2019) and patients can perceive distinct phosphenes (Zrenner et al., 2010; Lorach et al., 2015). Since the image is based on incident light entering the eye instead of an externally located camera for MPDAs with electronic amplification, patients can utilize natural eye movements. This is a marked advantage over camera-based visual prostheses, which are restricted to head movements for environmental scanning. Moreover, these MPDAs preserve the functionality of microsaccadic eye movements that prevent image fading by moving stimuli into and out of adjacent neurons’ receptive fields. The resultant spatial and temporal signal summation may result in more intelligible and naturalistic percepts; patients with photosensitive implants are capable of immediately recognizing shapes without any image processing (Stingl et al., 2013). A 1500 pixel MPDA subretinal implant offers visual acuity restoration up to 20/546 (Stingl et al., 2013), roughly translating to such real-world abilities as identification of office supplies and distinguishing between kitchen cutlery (Stingl et al., 2012). Some patients are even able to differentiate between large alphabet letters and combine them into words (Zrenner et al., 2010; Stingl et al., 2013). A recent study incorporated a 378-pixel array, achieving the highest visual acuity to date: 20/460. Additional testing of letter recognition and reading has already shown promising preliminary results (Palanker et al., 2019). Since MPDAs rely upon light transmittal through the cornea and lens, those with conditions that obscure light passage would not be eligible for such implants.

Research into subretinal implants has proven some initial physiological limitations, though researchers are actively seeking innovative solutions. The uneven photoreceptor density within the retina presents a major challenge. Since the density of cone photoreceptors decreases with increasing retinal eccentricity, uniform phosphene generation throughout the visual field would require MEAs with varying electrode diameters and inter-electrode spacing. Novel MEA designs that promote glial and neuronal migration may allow for lower stimulation levels and more densely packed arrays (Butterwick et al., 2009; Spira and Hai, 2013). Due to the variable thickness (Shroff, 2011) and fragility (Colodetti et al., 2007) of the degenerating retina, such designs are also surgically preferable to mitigate device-tissue contact. This prosthetic design has rehabilitative potential for millions of blind and visually-impaired individuals suffering from outer-retinal pathologies.

#### Epiretinal

Epiretinal prostheses interact with retinal ganglion cells. Owing to their downstream placement, epiretinal prostheses have a wider therapeutic potential than subretinal prostheses. Since extended periods of no photoreceptor input may cause signal corruption and intra-retinal neuronal degeneration, epiretinal prostheses may be the preferred visual prostheses for mid-to-late stage outer retinal pathologies. Epiretinal arrays can even selectively stimulate ganglion cells or bipolar cells based on stimulation parameters, including pulse polarity and duration (Boinagrov et al., 2014). Optimization of these features can reduce functional threshold levels, thus allowing for smaller electrode-diameters and denser MEAs before surpassing physiological safety limits. The implants are located adjacent to the spacious vitreous humor. This allows for larger electrical componentry and mitigates electrically-induced heat absorption by nearby tissue. However, unlike subretinal MEAs that are held in place by the underlying retinal pigment epithelium, the positioning of epiretinal arrays requires scleral-retinal tacks to achieve long-term perceptual consistency. Stabilization by an individual tack induces localized damage at the tack site and can physically separate distal regions of the array from the tissue surface (Majji et al., 1999; Mahadevappa et al., 2005). Currently there is an FDA-approved retinal prostheses (Luo and Da Cruz, 2016) which consists of a 60-electrode epiretinal device that has yielded patient improvements in spatial motor tasks (Ahuja et al., 2011), motion detection (da Cruz et al., 2016), and letter-reading performance (Da Cruz et al., 2013).

### Optic Nerve

Optic nerve prostheses can be efficacious for patients exhibiting retinal-based diseases or retinal detachment. Borrowing from peripheral-nerve stimulation technology (Mortimer et al., 1995), self-sizing cuff electrodes with four equidistant 200 μm^2^ contacts have been implanted around the optic nerve of a human patient (Veraart et al., 1998). By varying stimulation parameters, such as pulse duration and pulse train frequency optic nerve prostheses can elicit phosphene clusters of different sizes and in various locations (Veraart et al., 2003; Brelén et al., 2005). Although selective phosphene generation is rather crude, an implanted patient successfully localized, discriminated between and grasped small specific objects (Duret et al., 2006). Moreover, optic nerve prostheses benefit from enhanced electrode-phosphene efficiency and reduced tissue damage unlike high-density arrays. Such prostheses, however, are restricted to serial stimulation (Brelén et al., 2005) and lack adjustable phosphene luminosity (Delbeke et al., 2003), a feature positively correlated with performance scores on object discrimination tasks. The surface-based electrode contacts also increase current injection thresholds and reduce nerve fiber selectivity – the 1.2 million, 1-μm diameter optic nerve fibers already makes targeting specific points in the visual field extremely challenging (Jonas et al., 1992). In an effort to enhance fiber selectivity, penetrating electrode arrays have been inserted into the optic nerve. These MEAs can elicit cortical responses in animal models (Chai et al., 2008; Li et al., 2008; Lu et al., 2013; Gaillet et al., 2019) and one penetrating array, with wire electrodes progressing through the optic disk and into the optic nerve, has been implanted in a human patient (Sakaguchi et al., 2009). Similar to retinal prostheses, optic nerve prostheses, both surface-based and penetrating, benefit from a relatively less invasive intraocular surgery.

### Lateral Geniculate Nucleus (LGN)

At the optic chiasm, optic nerve fibers associated with the nasal half of each retina decussate and project to contralateral subcortical structures. Ninety percent of the retinal ganglion axons synapse at the dorsal lateral geniculate nucleus (LGN) of the thalamus (Kandel et al., 2000). An LGN prostheses has rehabilitative potential for individuals with either retinal or optic nerve pathologies.

Unlike the retina, LGN receptive fields have a consistent spatial density regardless of their visual field eccentricity. Since 60% of the LGN volume is devoted to processing the central 3° of the visual field (Schneider et al., 2004), lower-density MEAs could be used, reducing tissue damage due to mechanical insertion or electrical current delivery. Thalamic visual prostheses would require numerous electrodes to generate discrete phosphenes. One proposed method to facilitate high-density MEA uses a microwire bundle inserted via a cannula (Pezaris and Eskandar, 2009). Once the electrode nears the LGN, the microwires splay outward through the end of the cannula and penetrate the tissue at distinct locations. A model of bilateral 400-electrode implants is estimated to provide visual acuity up to 20/240 (Kyada et al., 2017). In order for such a device to be efficacious, electrode material, insertion speed and current injection levels must be optimized. As with retinal and optic nerve prostheses, intervention timing will be a key consideration to mitigate downstream neuronal degradation. Patients with severe glaucoma, for example, can show a significant progressive reduction in LGN size (Gupta et al., 2009). Although prostheses targeting the LGN have yet to be implanted in humans, animal models demonstrate device efficacy via cortical responses (Panetsos et al., 2011) and crude resolution via object localization tasks (Pezaris and Reid, 2007).

### Cortex

**FIGURE 1:**
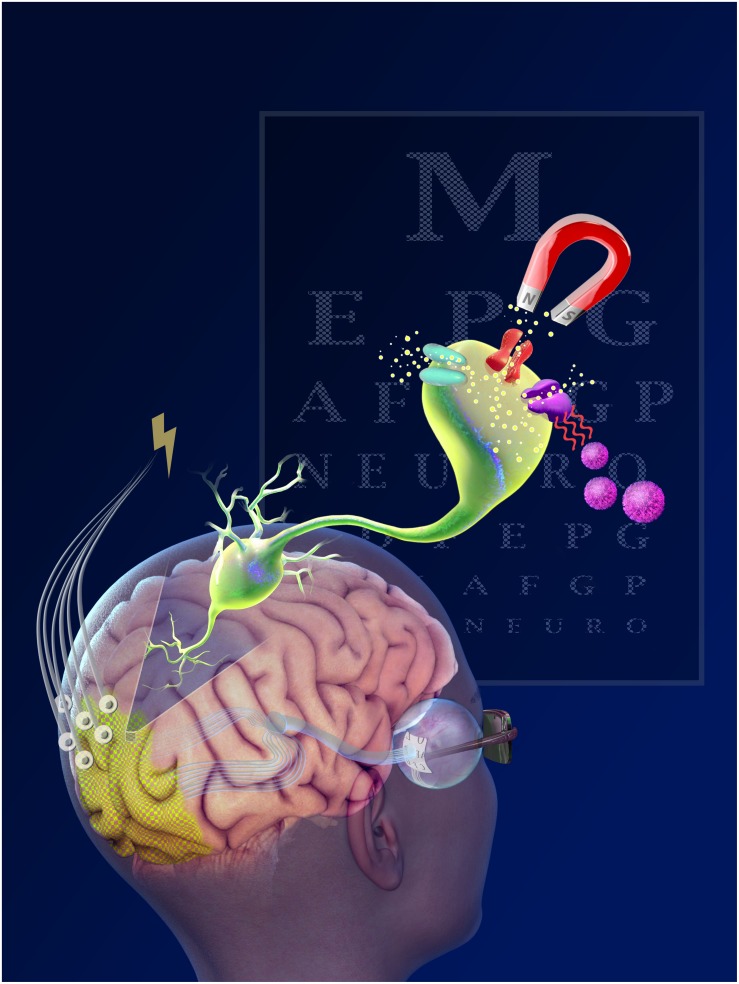
Electrical, light and magnetic stimulation for interfacing visual processing.

One of the most important features of cortical prostheses is the downstream location. This offers rehabilitative potential for blind and visually-impaired individuals for which a retinal, optic nerve or LGN visual prostheses would be ineffective (Gabel, 2016). Furthermore, cortical implants have the longest window for therapeutic intervention; instead of total neural degeneration, post-injury compensatory plasticity mechanisms recruit deafferented neurons from other cortical regions, offering the possibility of stimulation well beyond the onset of injury or disease (Sadato et al., 1996; Pietrini et al., 2004). After the LGN, the optic radiations transmit signals to layer 4 of the primary visual cortex (V1). Neurons with similar receptive fields are organized into 1 mm^2^ columns, which can be further subdivided into smaller columns responsive to orientation axis, color and ocular dominance. Similar to the LGN, these columns maintain a fairly consistent spatial density across the surface of V1 owing to the cortical magnification of central visual fields (Daniel and Whitteridge, 1961; Tehovnik, 1996; Dagnelie, 2011). Figure 1 illustrates the various interfaces and their location.

The subdural electrodes used in preliminary cortical visual prostheses proved capable of eliciting phosphenes but the substantial electrode-neuron distances required milliampere-range current injection levels (Brindley and Lewin, 1968; Dobelle et al., 1974, 1976). In the 1990s, penetrating intracortical electrodes were found to exhibit vastly superior spatial resolution and induce percepts with electrical currents two to three orders of magnitude less than those of surface electrodes (Bak et al., 1990; Schmidt et al., 1996).

Cortical prostheses can comfortably access central receptive fields, which are located near the surface of the occipital lobe, but face difficulty when targeting regions corresponding to peripheral fields. The interhemispheric fissure presents an anatomical barrier for stimulating roughly 85% of V1 (Trobe, 2001) and convolutions on the surface of the brain can bury receptive fields. However, since spatial representation is preserved and repeated multiple times across the visual cortex (Wandell et al., 2007), additional MEAs can be implanted in higher visual areas if receptive fields are inaccessible in lower regions (Dagnelie, 2011). Tertiary visual areas exhibit highly specific stimulus responsivity offering the potential for generating complex visual percepts. Early simulations for restoring functional vision via cortical prostheses estimate a total of at least 625 discrete phosphenes (Cha et al., 1992). In addition to electrode manufacturing, electronics for power and information transmission and processing must be developed. For example, one complementary metal-oxide-semiconductor chip can drive 473 electrodes independently (Wong et al., 2019), far more than is currently necessary for cortical-based MEAs.

The rapid advancement of microelectronic device fabrication and information processing has made cortical prostheses a viable rehabilitative option. There are several ongoing clinical trials testing the effectiveness and the risk associated with these implants (Fernández and Normann, 2017; Lowery et al., 2017; Troyk, 2017; Pouratian et al., 2019).

Next-generation pattern-recognition and deep-learning algorithms, especially those being investigated for computer vision will complement the complex response properties of visual areas in the brain. This could greatly expand cortical prostheses recipients’ capabilities, offering depth-perception, color discrimination, figure-ground discernment, and attentional modulation.

## Optogenetics

A number of organisms express light-sensitive proteins (opsins) allowing them to perform vital functions, such as phototaxis (Nagel et al., 2002; Sineshchekov et al., 2002) and energy conservation (Ernst et al., 2013). Optogenetic technologies use viral vectors to deliver genes encoding for opsins into defined tissue regions and cell populations with excellent temporal and spatial resolution (Boyden et al., 2005; Han and Boyden, 2007; Chow et al., 2010). Once the proteins are expressed, the cell can be controlled using a specific wavelength of light. Though the majority of optogenetic-based research has utilized microbial opsins, animal opsins have also been explored for vision restoration. Preclinical trials have shown that most animal opsins exhibit excellent light sensitivity but poor temporal responsivity, compared to microbial opsins (Lin et al., 2008; Cehajic-Kapetanovic et al., 2015). However, a recent study utilized an alternative animal opsin to achieve high light sensitivity and quick response kinetics, both of which are imperative for visual prostheses (Berry et al., 2019). An opsin-free approach incorporates small light-sensitive molecules, deemed photoswitches, that can be bound to specific cellular proteins, such as ion channels. The physical conformation of photoswitches can be selectively altered by exposing the molecule to different wavelengths of light, causing neuronal excitation and inhibition. Intravitreal injections of photoswitch molecules have been found capable of restoring light-sensitivity in blind animal models (Caporale et al., 2011; Polosukhina et al., 2012).

The retina has constituted the majority of optogenetic-based visual research because of its accessibility and transparent nature (Bi et al., 2006). Since there are more than 60 cell types within the retina (Masland, 2012), different classes of microbial and animal opsins can be expressed in particular cell populations. By activating and silencing individual cell types, patients can experience more naturalistic visual percepts (Bi et al., 2006; Lagali et al., 2008; Busskamp et al., 2010). Optical-based stimulation technologies, including μLED matrices (Wu et al., 2015; Khan et al., 2018) and computer-generated holography (Lutz et al., 2008; Reutsky-Gefen et al., 2013; Hernandez et al., 2016; Shemesh et al., 2017), are currently being developed to enable cellular and sub-cellular spatial resolution.

Preclinical studies have yielded promising results in non-human primates (Ivanova et al., 2010; Chaffiol et al., 2017) and clinical trials for patients with a variety of retinal-based diseases are currently underway (ClinicalTrials.gov NCT02556736, NCT03293524, and NCT03326336). Though biocompatibility concerns are minimal compared to electrode-based prostheses, clinical trials will determine if any long-term immune responses are present (Busskamp et al., 2012). A limitation of any light-based modality is its restricted penetration depth through tissue, which often requires the insertion of optical fibers. Fortunately, experiments involving non-human primates have shown that optogenetic stimulation of cortical neurons can induce visual percepts without the need to displace neural tissue (Jazayeri et al., 2012; Ju et al., 2018). Red-shifted opsins have been developed to mitigate tissue-induced light scattering as well as enable deeper stimulation and inhibition capabilities (Lin et al., 2013; Chuong et al., 2014). With the success of optogenetic-based therapies in preclinical models, it is likely that clinical trials will soon follow suit. The incorporation of these opsins may allow for the control of retinal or cortical neurons while minimizing the tissue damage characteristic of penetrating devices.

## Magnetic Stimulation

Magnetic fields pass through biological tissue with minimal reduction in field strength, making them desirable for non-invasive neurostimulation. Indeed, scientists have been experimenting with magnetically-induced phosphenes since the late 1800s (d’Arsonval, 1896).

The first transcranial magnetic stimulation (TMS) device was demonstrated in 1985 (Barker et al., 1985), and quickly became an important clinical and preclinical method for stimulating specific regions in the brain and spinal cord. TMS involves running an alternating current through a wire coil that is placed over a region of interest. Electromagnetic induction generates currents that are capable of stimulating neurons within the specific region. Since non-invasive stimulation of the brain reduces the risks encountered in surgical patients, such as hemorrhage, infection, and the overall cost of the procedure, TMS has recently gained interest for use in functional and behavioral research as well as rehabilitation research after brain injury (Ferbert et al., 1992; Celnik et al., 2009; Lu et al., 2015; Shin et al., 2018; Krishnan et al., 2019).

Cortical electromagnetic-based prostheses would benefit from superior coil-neuron proximity, permitting numerous small, low-power devices. Recent developments in the construction of micro-scale magnetic coils have the potential to increase the effectiveness and specificity of TMS. A number of micro-coils have demonstrated device efficacy *in vitro* (Bonmassar et al., 2012; Lee and Fried, 2016; Rizou and Prodromakis, 2018) and *in vivo* (Park et al., 2013; Minusa et al., 2017). While one major attraction of such devices is their non-invasive nature, device insertion can reduce coil-neuron distance, perceptual thresholds and necessary power input (Lee et al., 2016). Advancements in material sciences can accelerate the development of implantable micro-coils that are completely encased in biocompatible polymers to reduce electrically-induced tissue inflammation and glial scarring at the tissue-electrode interface. Additionally, the asymmetric current distribution permits selective activation of longitudinally-aligned axons (Bonmassar et al., 2012), adding a slight degree of specificity based on cellular orientation. One major limiting factor of micromagnetic stimulation is that conventional solenoid coils exhibit poor power efficiency and significant heat dissipation. Efforts into coil optimization may result in stronger magnetic flux densities and induced electric current at specific regions of interest (Bonmassar et al., 2014).

## Nanoparticle-Based Stimulation

Nanoparticles are extensively utilized in the fields of drug delivery (Kumari et al., 2010), biosensing (Doria et al., 2012) and tissue imaging (Gilad et al., 2008). Recently, ferrite-based magnetic nanoparticles were attached selectively to ion channels. Applying a magnetic gradient generates a force on the nanoparticles that most likely induces a conformational change on the associated membrane channel. This method has demonstrated to induce changes in cellular activity *in vitro* upon applying an external magnetic field (Hughes et al., 2007; Tay et al., 2016).

Another method is based on magnetic hyperthermia. When exposed to an alternating field, the orientation of a nanoparticle’s magnetic domain oscillates in accordance with the applied frequency. In response to weaker magnetic fields, this causes the nanoparticle to rotate and the particle-medium friction dissipates heat. With sufficient frequency, the localized heating can induce neuronal activation or inhibition via TRPV1 (Huang et al., 2010; Stanley et al., 2012; Chen et al., 2015; Munshi et al., 2017) or TMEM16A (Munshi et al., 2018) temperature-sensitive ion channels, respectively.

Voltage-gated ion channels expressed in V1 neurons can also be targeted by magnetoelectric composite nanoparticles. These nanoparticles, consisting of a magnetostrictive core and a piezoelectric shell, exhibit significant elastic coupling and magnetoelectric output (Nan et al., 2008). The magnetostrictive material can amplify a slowly varying external magnetic field, which induces a localized electric field via the piezoelectric compound. Rodent EEG recordings suggested that magnetoelectric nanoparticles offer an efficient method of neurostimulation (Guduru et al., 2015).

Nanoparticle materials are only suitable candidates for neurostimulation if they exhibit minimal cytotoxicity; little is known about the long-term health effects of nanoparticle delivery to the brain. Additionally, the method of effectively delivering nanoparticles to the central nervous system is a significant concern. Only a fraction of intravenously-injected nanoparticles successfully reach the brain and only do so by long-term endothelial cell endocytosis or by destroying their cellular membranes (Calvo et al., 2001; Yarjanli et al., 2017). However, non-invasive intranasal delivery may expedite delivery and reduce cell damage by bypassing the blood-brain barrier, which will make it more suitable for potential clinical applications.

## Genetically Encoded Magnetic Stimulation

Ongoing efforts have been dedicated to developing genetic-based neuromodulation technologies relying on magnetic changes. Magnetogenetics is a technology that allows cell, temporal, and location specific activation via magnetic fields and could evolve into a powerful non-invasive and effective technique for neurorehabilitation and vision restoration. These technologies mitigate concerns over cytotoxicity and tissue damage because no introduction of synthetic materials is required. A recent work showed the development of a construct encoding for ferritin protein subunits fused to TRPV4 receptors and its effectivity in inducing neural changes (Wheeler et al., 2016). However, neuronal response times were on the order of 20–60 s. Therefore, this approach may be effective for visual rehabilitation once allowing short responses time.

Several organisms including birds (Wiltschko and Wiltschko, 2005), fish (Quinn, 1980) and bacteria (Fassbinder et al., 1990), are known to rely on the Earth’s magnetic field for navigation and detection of prey and predators. Indeed, magnetic stimulation has been shown to trigger neural responses in the glass catfish, *Kryptopterus bicirrhis* (Lissmann and Machin, 1963; Struik et al., 2001). The magnetically sensitive gene has been identified and cloned, and was termed the electromagnetic-perceptive gene (EPG). *In vitro* and *in vivo* animal studies demonstrated that EPG is capable of eliciting neural responses (Krishnan et al., 2018). A number of research teams are working toward discovering the molecular structure and the signal transduction basis of this phenomenon. This will serve to expedite the optimization of stimulation parameters, including the strength and frequency of the applied magnetic field, as well as the EPG itself via artificial and targeted mutations.

## Sensory Substitution

Stimulation devices that convert optical information into tactile and auditory sensory inputs may offer alternative sight to individuals who are visually impaired due to stroke or brain injury (Amedi et al., 2001; Poirier et al., 2006). Tactile sensory substitution systems consisting of fingertip pin arrays that vibrated according to incoming signals from a video camera were developed in the 1960s and offered limited reading capabilities (Linvill and Bliss, 1966; Bliss et al., 1970). Other devices that stimulated the patients’ back extended such abilities to object recognition and differentiation (Bach-y-Rita et al., 1969; Collins, 1970).

The tongue is an excellent location for a sensory substitution device because it is densely innervated and coated in electrolytic saliva. With a tongue array consisting of over 100 electrode contacts, subjects significantly increased their visual acuity (Sampaio et al., 2001; Chebat et al., 2007). One drawback to positioning an electrode grid on the surface of the tongue, is that its usage would restrict normative tasks, such as conversing and eating. Stimulating gloves (Meers and Ward, 2005), headbands (Kajimoto et al., 2006), vests (Jones et al., 2006; Cancar et al., 2013), and belts (Van Erp et al., 2005) have also been explored for navigational, kinesthetic and vision reproduction purposes. The developments of methods such as electronic skin (Fu et al., 2018) will open new frontiers in tactile-based sensory substitution devices.

Other sensory substitution research has investigated auditory stimulation as a replacement for visual input. These devices use a number of translational parameters, including substituting pitch or frequency for vertical location, binaural intensity or time-scanning for horizontal position, loudness for brightness, and timbre for color (Meijer, 1992; Capelle et al., 1998; Abboud et al., 2014). While the development of visuoauditory systems initially lagged behind that of visuotactile, since its inception in the early 1990s (Meijer, 1992), such devices are now at the forefront of sensory substitution research. One reason for the popularization of visuoauditory devices is their increased absolute bandwidth over tactile devices; sighted individuals can recognize a total of 600 different tones (Capelle et al., 1998). Since blind individuals often exhibit enhanced auditory perception, it is feasible for the total number of tones to extend beyond the maximum estimated value. One limitation of these devices is that they restrict the ability to perceive auditory environmental cues, which is a highly preferred ability in blind individuals (Brewster and Brown, 2004).

## Conclusion

The total number of visual ailments is increasing rapidly among the general population in both developed and developing nations. Preliminary electrode-based visual prostheses demonstrated that they could induce visual percepts by interfacing with various regions of the visual system but lacked the efficiency to be adopted by blind and visually-impaired individuals in their everyday lives. Contemporary electrode-based visual prostheses have improved substantially since early prototypes. These devices can restore a number of abilities, such as crude object recognition and spatial navigation, and are now becoming a viable therapeutic consideration for blind and visually-impaired individuals. However, even with advanced micromachining and surgical procedures, limited spatial resolution and unavoidable tissue damage may render future electrode-based devices wanting. Nevertheless, non-electrode-based means for neurostimulation are being pursued. Optogenetic technologies introduces light-responsive proteins that can be used to excite or inhibit neural activity. For example, optogenetic technologies that introduce light-responsive proteins offer excellent resolution but cortical applications are restricted by fiber implantation and tissue damage. Other stimulation modalities, such as magnetic fields, have been explored to achieve non-invasive neuromodulation. Miniature magnetic coils are currently being developed to activate select groups of neurons. The poor coupling efficiency between magnetic fields and biological tissue leads to increasing power requirements and reducing achievable resolution. Magnetically-responsive nanoparticles or exogenous proteins can significantly enhance the coupling between external electromagnetic devices and any neurons affiliated with these modifications. The need to minimize cytotoxic effects for nanoparticle-based therapies will likely restrict the number of usable materials. Nevertheless, advances in identifying and utilizing proteins that respond to magnetic fields may lead to non-invasive, cell-specific stimulation and may overcome many of the limitations that currently exist with other methods. Finally, sensory substitution systems also serve as viable visual prostheses by converting visual input to auditory and tactile stimuli.

## Author Contributions

AF and GP conceptualized the manuscript, drafted the manuscript, revised and finalized it, and agreed to be accountable for all aspects of the work in ensuring that questions related to the accuracy or integrity of any part of the work are appropriately investigated and resolved.

## Conflict of Interest

The authors declare that the research was conducted in the absence of any commercial or financial relationships that could be construed as a potential conflict of interest.
